# Strontium isotope analysis of otoliths reveals differences in the habitat salinity among three sympatric stickleback species of the genus *Pungitius*

**DOI:** 10.1002/ece3.10463

**Published:** 2023-09-03

**Authors:** Konomi Uji, Asano Ishikawa, Ki‐Cheol Shin, Ichiro Tayasu, Jun Kitano

**Affiliations:** ^1^ Center for Ecological Research Kyoto University Otsu Japan; ^2^ Ecological Genetics Laboratory National Institute of Genetics Mishima Japan; ^3^ Research Institute for Humanity and Nature Kyoto Japan; ^4^ Present address: Department of Integrated Biosciences Graduate School of Frontier Sciences, The University of Tokyo Kashiwa Japan

**Keywords:** ear bone, elemental analysis, Gasterosteidae, ninespine stickleback, otolith microchemistry, salt concentration

## Abstract

The analysis of otolith Sr isotope ratios (^87^Sr/^86^Sr) is a powerful method to study fish migration in freshwater areas. However, few studies have applied this method to study fish movement in brackish‐water environments. Furthermore, despite the fact that habitat differentiation has been shown to drive genetic differentiation and reproductive isolation among stickleback fish, no studies have used the otolith ^87^Sr/^86^Sr ratios to analyze habitat differentiation between stickleback ecotypes and species. In this study, we analyzed the otolith ^87^Sr/^86^Sr ratios of three sympatric stickleback species of the genus *Pungitius* in the Shiomi River on Hokkaido Island, Japan: *P. tymensis*, the brackish‐water type of the *P. pungitius–P. sinensis* complex, and the freshwater type of the *P. pungitius–P. sinensis* complex. First, we created a mixing equation to depict the relationship between habitat salinity and the ^87^Sr/^86^Sr ratios of river water. We found that the otolith ^87^Sr/^86^Sr ratios differed significantly among the three species, indicating that the three species utilize habitats with different salinities: *P. tymensis* and the brackish‐water type inhabit freshwater and brackish‐water environments, respectively, with the freshwater type using intermediate habitats. In addition, we found that some freshwater individuals moved to habitats with higher salinities as they grew. Our study demonstrates that the analysis of otolith ^87^Sr/^86^Sr ratios is a useful method for studying the habitat use of fish in brackish‐water environments and habitat differentiation among closely related sympatric and parapatric species.

## Graphical abstract

We analyzed the otolith Sr isotope ratios of three sympatric stickleback species of the genus *Pungitius* in the Shiomi River on Hokkaido Island, Japan. We created a mixing equation to depict the relationship between habitat salinity and the Sr isotope ratios of river water. We found that the otolith Sr isotope ratios differed significantly among the three species, indicating that the three species utilize habitats with different salinities.

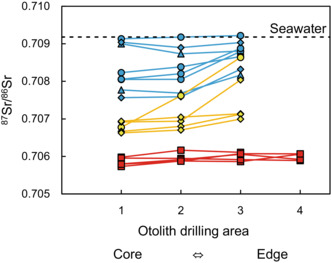

## 1 INTRODUCTION

Various methods have recently been developed for the analysis of fish migration with the aim of ecological research, fishery resource management, and conservation (Cooke et al., 2022; Lucas et al., 2001). For example, technologies for tagging fish using acoustic and radio transmitters are greatly advancing (Hussey et al., 2015). However, it remains difficult to physically attach tags to small fish. Otolith microchemistry analysis is another method of analyzing fish migration that enables us to reconstruct the past habitat of an individual fish by analyzing elements in otoliths because otoliths are metabolically inert and continuously grow from the core to the edge (Campana, 1999; Campana & Thorrold, 2001; Elsdon et al., 2008; Elsdon & Gillanders, 2003). Otolith microchemistry is especially useful for analyzing the past migration and habitats of small or juvenile fish (e.g., Arai & Goto, 2005; Arai et al., 2020; Hamman & Kennedy, 2012; Muhlfeld et al., 2012).

The strontium to calcium concentration ratio (Sr/Ca) has been used to study the migratory behavior of diadromous fish in habitats with different salinities (Arai et al., 2020; Walther & Limburg, 2012). The otolith Sr/Ca ratios in fish are positively correlated with those in ambient water. Furthermore, the Sr/Ca ratios of freshwater habitats are much lower than those of marine habitats in most geographical regions. Therefore, changes in the otolith Sr/Ca ratios along the direction of otolith growth, which indicates the age of the fish, can be used to investigate whether and when fish migrate between freshwater and seawater (Walther & Limburg, 2012). However, otolith Sr/Ca ratios are determined not only by ambient water Sr/Ca ratios but also by factors that influence the regulation of elemental uptake and assimilation processes, such as temperature (Campana, 1999; Elsdon & Gillanders, 2003). These confounding effects will make it difficult to reconstruct the migration history of fish between habitats with small differences in Sr/Ca ratios (e.g., within brackish‐water habitats) by analyzing otolith Sr/Ca ratios.

Compared to Sr/Ca ratios, otolith ^87^Sr/^86^Sr ratios generally reflect the ^87^Sr/^86^Sr ratios of ambient water better because ^87^Sr/^86^Sr ratios are not altered by the physiological process of uptake and assimilation into the body (Barnett‐Johnson et al., 2008; Kennedy et al., 1997, 2000; Muhlfeld et al., 2012). In addition, seawater ^87^Sr/^86^Sr ratios are globally constant (Faure & Mensing, 2005), whereas ^87^Sr/^86^Sr ratios of river water differ regionally, mainly reflecting differences in bedrock geology (Barnett‐Johnson et al., 2008; Bataille et al., 2014; Bataille & Bowen, 2012). Therefore, the analysis of otolith ^87^Sr/^86^Sr ratios can be used to reconstruct the migration history of fish between water bodies with different ^87^Sr/^86^Sr ratios (e.g., Brennan et al., 2015; Hamman & Kennedy, 2012; Kennedy et al., 2002). Although most previous studies using otolith ^87^Sr/^86^Sr ratios aimed to study fish migration within freshwater and between freshwater and seawater, few studies have attempted to identify the salinity ranges of fish habitats in brackish water (e.g., Hobbs et al., 2010), despite the fact that the ^87^Sr/^86^Sr ratios of brackish water contain information on the mixing ratio of freshwater and seawater and can be used as a proxy for salinity (Walther & Limburg, 2012).

Stickleback fish, *Gasterosteidae*, have been used as model organisms for behavioral, evolutionary, and ecological studies (Bell & Foster, 1994; Hendry et al., 2013; McKinnon et al., 2019; Merilä, 2013; Schluter, 2000; Tinbergen, 1951). Recently, the genetic mechanisms for freshwater adaptation and the convergent evolution of morphology and physiology have been extensively investigated in stickleback fish (e.g., Chan et al., 2010; Colosimo et al., 2005; Ishikawa et al., 2019; Kitano et al., 2009, 2010, 2012; McKinnon & Rundle, 2002; Peichel & Marques, 2017; Schluter, 2000). In particular, pairs of ecotypes that exploit contrasting habitats (ocean vs. freshwater, upstream vs. downstream in rivers, rivers vs. lakes, and offshore vs. coastal areas in lakes) provide great opportunities to investigate the ecological and genetic mechanisms of phenotypic divergence and reproductive isolation (Bell & Foster, 1994; McKinnon & Rundle, 2002; Schluter, 2000). However, with a few exceptions (Arai et al., 2003a, 2003b, 2010, 2020; Arai & Goto, 2005, 2008), most studies have examined habitat differentiation through capture or direct observations. The development of otolith microchemistry studies of sticklebacks will increase the quantity and quality of information regarding their habitat use in the field.

In this study, otolith ^87^Sr/^86^Sr ratio analysis was used to investigate the differences in the microhabitat and migration of three sympatric stickleback species belonging to the genus *Pungitius*. Three *Pungitius* species inhabit Hokkaido, Japan: *P. tymensis*, the brackish‐water type of the *P. pungitius–P. sinensis* complex, and the freshwater type of the *P. pungitius–P. sinensis* complex (Ishikawa et al., 2013; Takahashi et al., 2016; Takata et al., 1987). Previously, the species identification of the *P. pungitius–P. sinensis* complex was based on armor plate morphology, such that individuals with complete plates from the front to the back of the body were called *P. sinensis* and individuals with gaps in the middle of the armor plates were called *P. pungitius*. However, subsequent genetic studies have shown that such morphological species identification does not match the genetic clusters (Ishikawa et al., 2013; Takahashi et al., 2016; Yamasaki et al., 2020). Therefore, we refer to two genetically distinct species of the *P. pungitius–P. sinensis* complex as the brackish and freshwater types, according to previous studies (Ishikawa et al., 2013; Takahashi et al., 2016). The Shiomi River in eastern Hokkaido is a sympatric habitat for these three species. The salinity of the nesting sites of these three sympatric species is different (Tsuruta et al., 2008), as is their salinity tolerance (Ishikawa et al., 2013), suggesting habitat isolation. The brackish‐water type and *P. tymensis* were observed in the most downstream and upstream parts of the river, respectively, with the freshwater type in the middle part. However, because these studies only observed adult fish, we do not know whether they shift their microhabitats depending on their life stages.

Here, we tested whether otolith ^87^Sr/^86^Sr ratio analysis can be used to detect habitat use differences among these three sympatric species of the Shiomi River at different life stages. To this end, we first observed otolith morphology to determine how otoliths should be dissected for subsequent ^87^Sr/^86^Sr ratio analyses. We drilled several otolith areas using a micro‐mill, followed by thermal ionization mass spectrometry (TIMS) to detect differences in ^87^Sr/^86^Sr ratios. For otoliths to be drilled, three factors must be determined: the direction of otolith polishing, the position of drilling, and the depth of drilling. To compare otolith parts formed at the same time among individuals, we investigated the number and position of translucent rings on the otoliths to determine whether the translucent rings could be used as boundaries for drilling. Second, we investigated the relationships between ^87^Sr/^86^Sr ratios and salinity in the Shiomi River to examine whether ^87^Sr/^86^Sr ratios can be used as a proxy for salinity. Finally, we compared the otolith ^87^Sr/^86^Sr ratios to examine whether otolith ^87^Sr/^86^Sr ratios differ among three species at multiple life stages and converted otolith ^87^Sr/^86^Sr ratios into salinity using a mixing equation.

## 2 MATERIALS AND METHODS

### 2.1 Water and fish sampling

**FIGURE 1:**
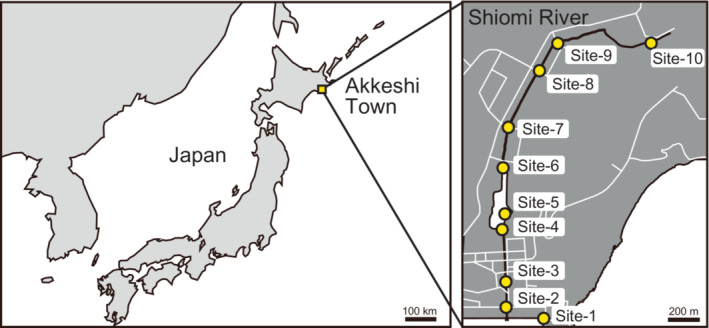
Location of sampling sites (10 sites) in the Shiomi River, Akkeshi Town, Hokkaido Prefecture, Japan (43°01′ N, 144°50′ E).

Sticklebacks and water were sampled from the Shiomi River, Akkeshi Town, Hokkaido Prefecture, Japan (43°01′ N, 144°50′ E; Figure 1). The Shiomi River is a short river with approximately 4 km of length in a watershed composed of a marsh and a single bedrock geology of marine conglomerate formed during the period from the Cenozoic Paleogene Paleocene Danian stage to the Eocene Ypresian stage (Figure S2, Geological Survey of Japan, AIST, 2022). In the Shiomi River, the salinity of the water increases gradually from upstream to downstream (Tsuruta et al., 2008). Water was sampled at 10 sites (Figure 1) at four time points (May 11–12, 2016, morning of July 3, 2016 at low tide, afternoon of July 3, 2016 at high tide, and May 28, 2018) to examine not only the geographical variations but also the annual, seasonal, and diurnal changes (i.e., low tide vs. high tide) in salinity and isotope ratios of water. In the field, water was filtered through a disposable cellulose acetate filter with a pore size of 0.2 μm (DISMIC 25CS020AS, Advantec Toyo Kaisha) and subsequently stored in 50‐mL polyethylene bottles. Before use, the bottles were rinsed three times with a few milliliters of filtered water. After the water samples were transported to the laboratory, they were stored at 5°C until further use. For all the water samples, salinity was measured as conductivity using a portable electrical conductivity meter (LAQUAact ES‐71; HORIBA).

Fish were sampled at four sites (Figure 1: site‐6, site‐8, site‐9, and site‐10) on April 18–20, 2017, using minnow traps set on the river bottoms. *P. tymensis* and other species can be easily distinguished based on their external morphology. *P. tymensis* has a higher caudal peduncle and shorter dorsal spines than the other two species (Ishikawa et al., 2013). The brackish‐water and freshwater types were distinguished with microsatellite markers (see Section 2.2). The brackish‐water type was sampled from site‐6 (*n* = 3), site‐8 (*n* = 8), and site‐9 (*n* = 4); the freshwater type was sampled from site‐8 (*n* = 2) and site‐9 (*n* = 5); and *P. tymensis* was sampled from site‐10 (*n* = 9) (Table A1). Sagittal otoliths were extracted from the fish in the field. The extracted otoliths were transported to the Research Institute for Humanity and Nature (RIHN) and washed in an ultrasonic cleaner with ultrapure water, dried at 60°C, and then weighed with an ultra‐microbalance (XPR2UV; Mettler‐Toledo International). After weighing, the otoliths were stored in a room maintained at 20°C until polishing.

### 2.2 Genetic analysis

Genomic DNA was isolated from the pectoral fins using the DNeasy Blood & Tissue Kit (Qiagen). Fish were genotyped using eight microsatellite markers (*Pun212*, *Pun134*, *Pun171*, *Pun19*, *Pun68*, *Pun117*, *Stn433*, and *Pun78*), which were shown to follow the Hardy–Weinberg equilibrium and are useful for species identification (Ishikawa et al., 2013). The experiments and analyses were conducted as previously described (Ishikawa et al., 2013). Briefly, PCR products were amplified using a KAPA2G Fast Multiplex PCR Kit (KAPA Biosystems) and run at the Genotyping Center of BEX Co., Ltd. Fragment lengths were determined using Peak Scanner Software (Life Technologies). Using the STRUCTURE software (Pritchard et al., 2000) with *K* = 2, we calculated the probability of assignment to one of the two genetic clusters after 200,000 iterations with a burn‐in of 25,000 iterations (Figure S1; Table S1). Eleven samples of the freshwater type and 44 samples of the brackish‐water type used by Ishikawa et al. (2013) were also added to the STRUCTURE analysis to determine which genetic cluster corresponded to which species.

### 2.3 Polishing and drilling otoliths

**FIGURE 2:**
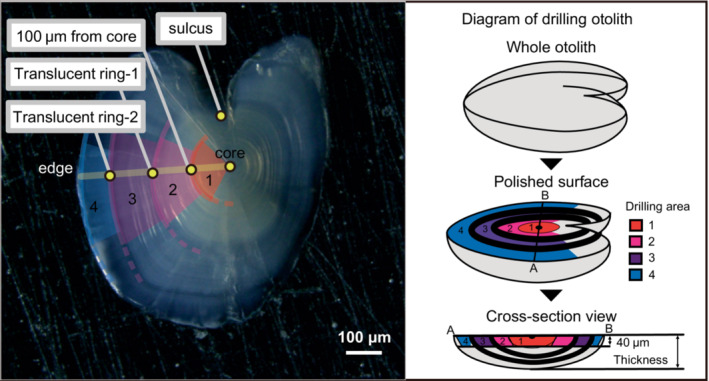
An image of an otolith with clear translucent rings. The positions of the translucent rings of otoliths were measured from the core toward the longer end along with the line perpendicular to the sulcus (yellow line). Drilling areas are shown in the image: (1) core–100 μm (red), (2) 100 μm–“translucent ring‐1” (pink), (3) “translucent ring‐1”–“translucent ring‐2” or edge (purple), (4) “translucent ring‐2” –edge (blue).

**FIGURE A1:**
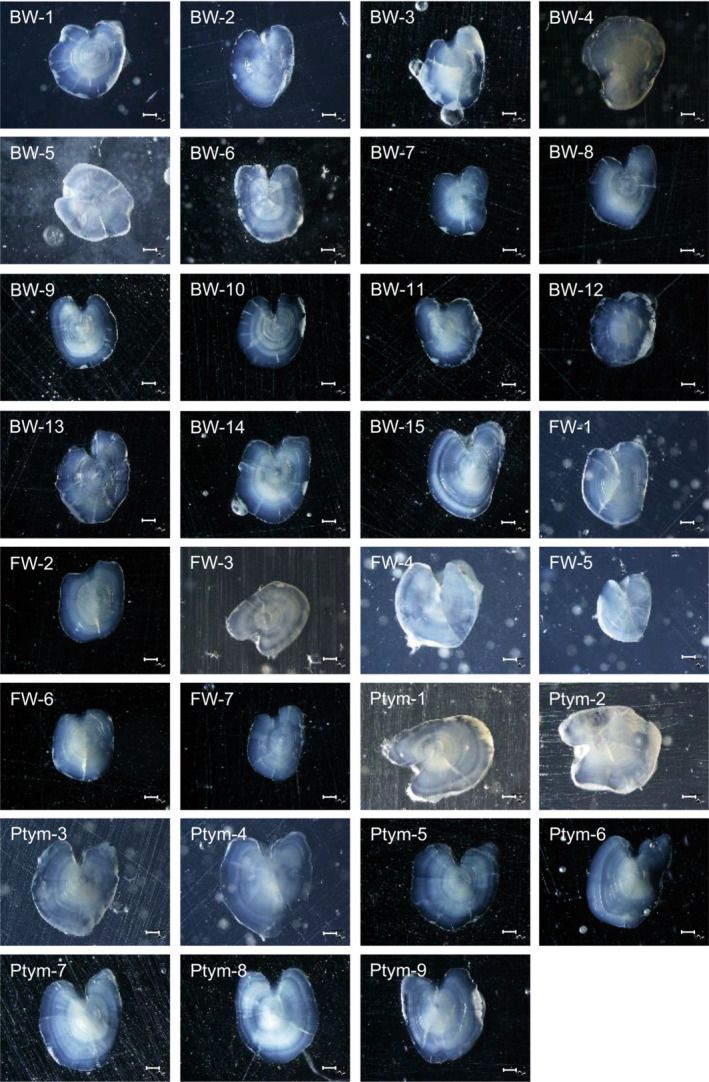
Images of all otoliths. Bar = 100 μm.

All otoliths were embedded in epoxy resin and polished using a polishing machine (IM‐P2; IMT Co.) and abrasive papers until each core appeared (Figure 2). The otoliths were polished in the sagittal plane (Secor et al., 1991) as in previous studies (e.g., Arai & Goto, 2008). The otoliths were observed, and images were captured using a polarizing microscope (BX51; Olympus) with a digital camera (DP20; Olympus) (all images are shown in Figure A1). The number and positions of the translucent rings (Figure 2) of otoliths in these images were measured using ImageJ (Rasband, 1997–2023) to determine the drilling areas. Most of the otoliths were opaque, with several translucent rings. To determine the boundaries of the drilling areas, we measured the positions of the translucent rings. There were three types of otoliths. For otoliths with clearly visible translucent rings, we measured the position of each translucent ring as the distance from the core along the line perpendicular to the sulcus of the otoliths toward the longer side (Figure 2). Second, for some otoliths, several translucent rings were vague and/or multiple rings were present. In these cases, we considered the positions of the translucent rings nearest the mean positions of the translucent rings calculated from otoliths with clearly visible rings (see above) as the boundaries. Finally, some of the otoliths lacked clear rings near their mean positions. We did not determine the ring positions for these otoliths. Instead, the midpoints between the anterior and posterior rings were used as boundaries for the drilling areas. Five otoliths had another thin translucent ring inside their first translucent ring. The mean position of this thin ring was approximately 100 μm from the core (see Section 3); therefore, we used this position (100 μm from the core) as another boundary. The thicknesses of the polished otoliths were measured to determine the drilling depth (see Section 3 below).

Some of the polished otoliths (brackish‐water type: *n* = 9, freshwater type: *n* = 5, and *P. tymensis*: *n* = 5) were adhered to slide glasses, washed in an ultrasonic cleaner with ultrapure water, and dried at 60°C. The otoliths were drilled using an automated micro‐mill apparatus (GEOMILL326; Izumo Web) installed in a simple, clean booth, and the powdered otoliths were collected using ultrapure water in acid‐washed Teflon vials.

### 2.4 Analysis of the ^87^Sr/ ^86^Sr ratio by TIMS

All samples were moved to a clean room at the RIHN. Each collected otolith sample was completely dissolved in concentrated HNO_3_ (ultrapur‐100; Kanto Chemical Industry) at 80–120°C overnight on a hot plate. Water samples containing approximately 200 ng of Sr and the solutions obtained by dissolving the otoliths were evaporated. The residues were then dissolved in 3.5 N HNO_3_ and separated by using a Sr resin (particle sizes 100–150 μm; Eichrom Technologies) and MCI GEL CHP20P (particle sizes 75–150 μm; Sigma‐Aldrich) and a hand‐made perfluoroalkoxy alkane tube column. Purified Sr was loaded onto preconditioned tungsten filaments with a tantalum activator and measured in the single‐filament mode. ^87^Sr/^86^Sr ratios were measured using TIMS (TRITON; Thermo Fisher Scientific). The internal precision of the measurements (one standard error) of water and otoliths calculated from 100 or 150 repeated measurements per sample was within 9.6 × 10^−6^ and within 3.7 × 10^−5^, respectively (Tables A2 and A3). The intensities of the ^88^Sr beams of the water and otoliths throughout the analyses were approximately 3–5 V and 500 mV, respectively. The measured ^87^Sr/^86^Sr ratios were normalized to the measured ^86^Sr/^88^Sr ratios (^86^Sr/^88^Sr = 0.1194). The mean ^87^Sr/^86^Sr ratio of the standards (SRM 987; National Institute of Standards and Technology) throughout the analyses was 0.710257 ± 0.0000029 (SE, *n* = 39), and all measurements were normalized to ^87^Sr/^86^Sr = 0.71025, as recommended by Faure and Mensing (2005).

### 2.5 Statistical processing

The following two‐component (freshwater and seawater) mixing equation was used to investigate the relationship between ^87^Sr/^86^Sr ratios and the salinity of water in the Shiomi River.

(1) $$R_{\mathrm{mix}}=\frac{\left(R_{\mathrm{sw}}-R_{\mathrm{fw}}C\right)\times S_{\mathrm{mix}}+R_{\mathrm{fw}}C}{\left(1-C\right)\times S_{\mathrm{mix}}+C}$$

*R*_mix_: mixed water ^87^Sr/^86^Sr; *R*_sw_: seawater ^87^Sr/^86^Sr; *R*_fw_: freshwater ^87^Sr/^86^Sr; *C*: Sr concentration of freshwater divided by that of seawater; *S*_mix_: salinity of mixed water divided by 35‰. This equation was made by modifying the two‐component equation shown by Bryant et al. (1995) and assuming that the salinities of seawater and freshwater were 35‰ and 0‰, respectively. We substituted 0.70918 for *R*_sw_ based on the results of Faure and Mensing (2005). To estimate the *R*_fw_ and *C* values, the measured *R*_mix_ and *S*_mix_ values were fitted to Equation (1) by using non‐linear least squares; the “nls” function in R Ver 4.3.1 was used (R Core Team, 2023). The 95% confidence intervals (CI) of the equation estimated from all data were calculated using the “predictNLS” function in the R package “propagate” (Spiess, 2018).

Differences in the otolith ^87^Sr/^86^Sr ratios among species, drilling areas, and sampling sites were first tested using the Kruskal–Wallis test. When the Kruskal–Wallis test yielded significant results, the Steel–Dwass post hoc test was conducted to identify pairs that were significantly different. These tests were conducted using the statistical analysis software R (ver. 4.3.1) (R Core Team, 2023). The function “kruskal. test” of the Kruskal–Wallis test was used, while a custom R script from Aoki (2004) was used for the Steel–Dwass test. Differences among the drilling areas were tested separately for each species. The variation among sampling sites was compared only for the brackish‐water type because other species could not be collected from many different sites. The mean otolith ^87^Sr/^86^Sr ratios were converted into salinity by using a mixing equation estimated from all water data. The 95% CI for the converted salinity values was calculated by the “invest” function in the R package “investr” with the interval as “Wald” (Greenwell & Schubert Kabban, 2014).

## 3 RESULTS

### 3.1 Otolith structure

Our genetic STRUCTURE analysis confirmed that the two cryptic species, the brackish‐water and the freshwater types, were genetically differentiated (Figure S1). In the present study, no hybrids were found. Using these genetic data, we determined the species identification of the *P. pungitius*–*P. sinensis* species complex used for the subsequent otolith analysis.

Eleven of the 31 otoliths analyzed in this study had clearly visible translucent rings (*n* = 4 for the brackish‐water type, *n* = 4 for the freshwater type, and *n* = 3 for *P. tymensis*). One or two translucent rings were observed in these otoliths. Another thin translucent ring was observed inside the first translucent ring in five of the 11 otoliths. The translucent rings at the outermost edges of the otoliths were not counted (Figure 2; Figure A1). All data on the number and positions of the translucent rings of the otoliths are shown in Table A1. The mean position of the first rings was 171 ± 9 μm from the core, that of the second ring was 303 ± 10 μm (mean ± SE) from the core, and that of the thin translucent rings was approximately 100 μm from the core.

The thickness of the polished otoliths was 133 ± 4 μm (mean ± SE) (Figure 2; Table A1). All otoliths were drilled to a depth of 40 μm to obtain as much sample volume as possible and avoid contaminating other areas (see Figure 2).

### 3.2 Relationship between the ^87^Sr/ ^86^Sr ratios and salinity of water

**FIGURE 3:**
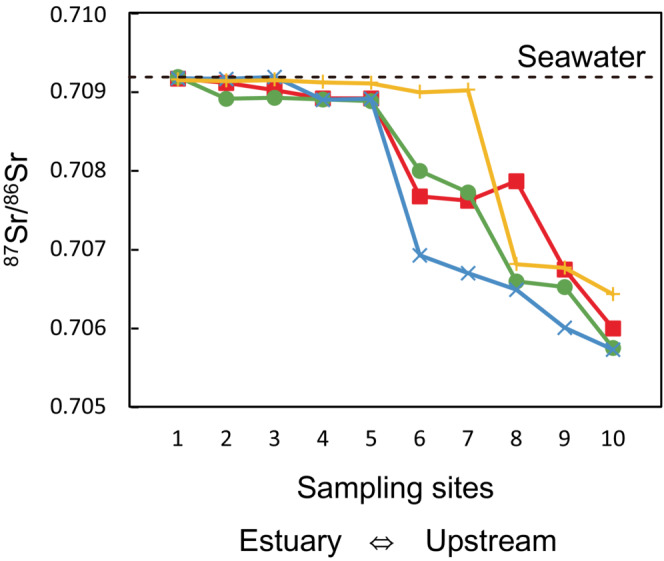
The ^87^Sr/^86^Sr ratios at 10 sampling sites. A dotted line indicates a seawater ^87^Sr/^86^Sr ratio of 0.70918 ± 0.00001 (Faure & Mensing, 2005). The red line and square plots indicate the data of May 11–12, 2016; the green line and circle plots indicate the data of the morning of July 3, 2016; the blue line and x‐cross plots indicate the data of the afternoon of July 3, 2016; the yellow line and cross plots indicate the data of May 28, 2018.

**FIGURE 4:**
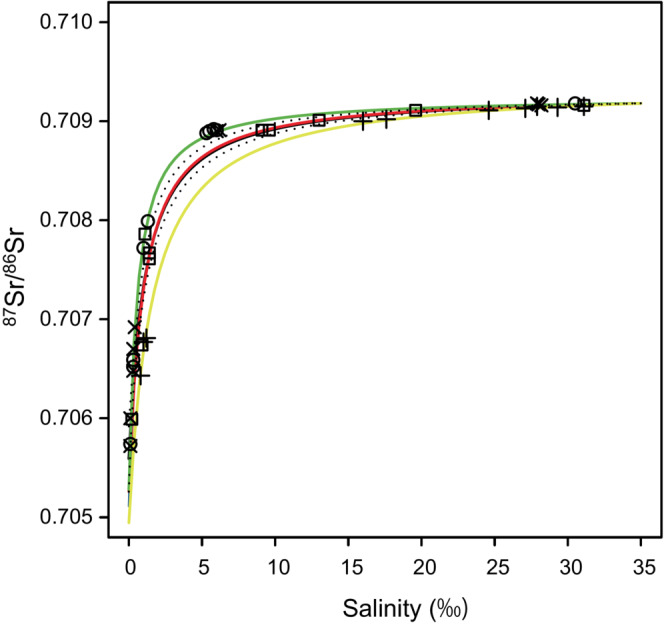
The relationship between water salinity and ^87^Sr/^86^Sr ratios. The *x*‐axis indicates water salinity, while the *y*‐axis indicates the water ^87^Sr/^86^Sr ratios. Plots of mixing equations are also shown. The black line indicates the mixing curve made with all data; the red line and square plots indicate the data of May 11–12, 2016; the green line and circle plots indicate the data of the morning of July 3, 2016; the blue line and x‐cross plots indicate the data of the afternoon of July 3, 2016; the yellow line and cross plots indicate the data of May 28, 2018. As the green and blue lines overlap, we cannot distinguish them in the figure. The dotted lines indicate the 95% CI of the equation estimated from all data.

The ^87^Sr/^86^Sr ratios and salinities of water are shown in Table A3. In the Shiomi River, the ^87^Sr/^86^Sr ratio decreased with the increasing distance of the sampling site from the estuary (Figure 3). The maximum geographical variation of ^87^Sr/^86^Sr ratios among sites was approximately 0.003, and the maximum annual, seasonal, and diurnal changes in ^87^Sr/^86^Sr ratios were approximately 0.001–0.002 (Figure 3). The water ^87^Sr/^86^Sr ratios tended to increase as salinity increased (Figure 4). The measured ^87^Sr/^86^Sr ratios and salinity were fitted to Equation (1) using the non‐linear least squares method to estimate the *R*_fw_ and *C* values. Using all the water data, it was estimated that *R*_fw_ was 0.70559 (SE:0.00016) and *C* was 0.031 (SE:0.0045). An analysis of data at each sampling time point separately showed that *R*_fw_ was 0.70526 (SE:0.00042) and C was 0.027 (SE:0.0066) on May 11–12, 2016, *R*_fw_ was 0.70511 (SE:0.00005), and *C* was 0.016 (SE:0.00054) in the morning of July 3, 2016, *R*_fw_ was 0.70528 (SE:0.00013) and *C* was 0.017 (SE:0.0020) in the afternoon of July 3, 2016, and *R*_fw_ was 0.70494 (SE:0.00030) and *C* was 0.042 (SE:0.0073) on May 28, 2018. There were apparent annual and seasonal changes, but no clear diurnal change in the mixing equation (Figure 4). The 95% CI of the equation estimated from all the data was illustrated in Figure 4.

### 3.3 Comparison of the otolith ^87^Sr/ ^86^Sr ratios

**FIGURE 5:**
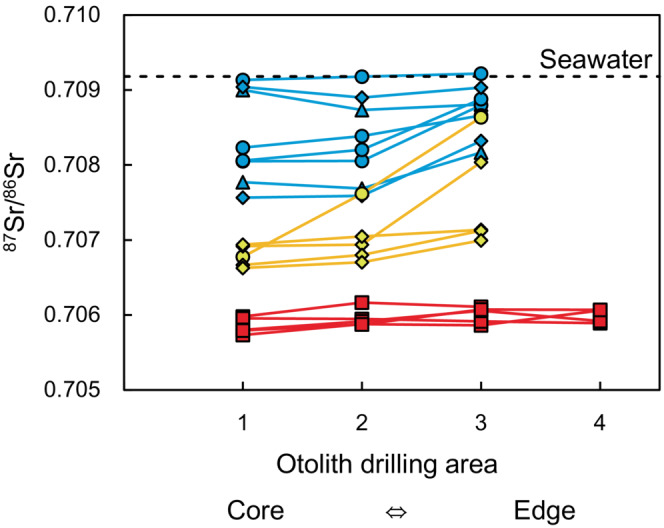
Otolith ^87^Sr/^86^Sr ratios in three *Pungitius* species. The *x*‐axis indicates the drilling areas of otoliths (see Figure 2 and the text for the drilling areas), while the *y*‐axis indicates the otolith ^87^Sr/^86^Sr ratios. Different colors indicate different species: blue, the brackish‐water type; yellow, the freshwater type; red, *Pungitius tymensis*. Different symbols indicate the sampling sites: triangle, site‐6; circle, site‐8; diamond, site‐9; square, site‐10. A dotted line indicates the seawater ^87^Sr/^86^Sr ratio, which is 0.70918 ± 0.00001 (Faure & Mensing, 2005).

The otolith ^87^Sr/^86^Sr ratios are shown in Figure 5 and Table A2. There were significant differences in the ^87^Sr/^86^Sr ratios of otoliths between species (Steel‐Dwass test, *p* < .001 for all species pairs). There were no significant differences in the otolith ^87^Sr/^86^Sr ratios between the drilling areas except between area‐1 and area‐3 in the freshwater type (Steel‐Dwass test, *p* = .024). There was no significant difference in the otolith ^87^Sr/^86^Sr ratios of the brackish‐water type among the sampling sites (Kruskal‐Wallis test, *p* = .056). The salinity of the habitats was inferred by converting the mean ^87^Sr/^86^Sr ratios using the equation estimated from all water data. The converted salinity values of area‐1, area‐2, and area‐3 of the brackish‐water type were 3.6‰ (95% CI = 0.61–6.6‰), 3.4‰ (95% CI = 0.65–6.1‰), and 6.7‰ (95% CI = −1.4–14.8‰), respectively. The converted salinity values of area‐1, area‐2, and area‐3 of the freshwater type were 0.53‰ (95% CI = 0.16–0.90‰), 0.70‰ (95% CI = 0.25–1.2‰), and 1.3‰ (95% CI = 0.50–2.1‰), respectively. The converted salinity values of area‐1, area‐2, area‐3, and area‐4 of *P. tymensis* were 0.08‰ (95% CI = −0.12–0.29‰), 0.12‰ (95% CI = −0.10–0.34‰), 0.14‰ (95% CI = −0.08–0.36‰), and 0.13‰ (95% CI = −0.09–0.35‰), respectively.

## 4 DISCUSSION

### 4.1 Association between the ^87^Sr/ ^86^Sr ratios and salinity of the Shiomi River water

The ^87^Sr/^86^Sr ratios of water varied among the sites in the Shiomi River, with higher ratios closer to the estuary (Figure 3). The water ^87^Sr/^86^Sr ratios also increased as water salinity increased (Figure 4), suggesting that water salinity is one of the factors determining the water ^87^Sr/^86^Sr ratios in this river. Given that the SE of ^87^Sr/^86^Sr ratios of each otolith sample was in the order of 10^−5^, we consider that geographical variations of water ^87^Sr/^86^Sr ratios (0.003) are large enough to analyze habitat differences in this river. However, it would be difficult to directly compare otolith ^87^Sr/^86^Sr ratios to water ^87^Sr/^86^Sr ratios at each site because of the large annual, seasonal, and diurnal changes in water ^87^Sr/^86^Sr ratios within the sites.

As the two‐component mixing equation for the ^87^Sr/^86^Sr ratio and salinity is theoretically curvilinear (Walther & Limburg, 2012), our data were also plotted as curvilinear (Figure 4). To rigorously examine the relationship between water ^87^Sr/^86^Sr ratios and salinity in the Shiomi River, we estimated the unknown parameters *R*_fw_ and *C* from the measured ^87^Sr/^86^Sr and salinity values. The *R*_fw_ values were estimated to be 0.70494–0.70528. The *R*_fw_ values are supposed to be ^87^Sr/^86^Sr ratios of water with 0‰ salinity, but because this value was unavailable, we assumed that *R*_fw_ was close to the ^87^Sr/^86^Sr ratios of river water. Nakano et al. (2020) created rough maps of the ^87^Sr/^86^Sr ratios of groundwater in Japan using the geostatistical tool ArcGIS. Our estimated *R*_fw_ values are similar to those shown on their maps (0.7048–0.7056), suggesting that our estimation was reasonable. The relatively conserved *R*_fw_ values estimated using different sampling time points may be due to the single bedrock geology in the Shiomi River (Figure S2). As Goldstein and Jacobsen (1988) reported that ^87^Sr/^86^Sr ratios of rivers and lakes in various regions of the world ranged from 0.7045 to 0.950, the Shiomi River has relatively low ^87^Sr/^86^Sr ratios compared to other regions.

*C* values estimated in this study ranged from 0.016 to 0.042. *C* values generally covary with the Sr concentrations in both freshwater and seawater. The Sr concentrations in seawater are relatively constant with little regional variability (de Villiers, 1999). In contrast, the Sr concentrations in river water can vary regionally by as much as three orders of magnitude depending on the bedrock type because Sr in river water is mainly derived from the bedrock (Walther & Limburg, 2012). Although the temporal variability of the Sr concentration in river water is small, its concentrations are affected to some extent by hydrological processes (Elsdon et al., 2008). Therefore, temporal variations in Sr concentrations in the water of the Shiomi River may have caused variations in *C* values.

Annual and seasonal changes in our mixing equation were mainly caused by variations in *C* values. The annual or seasonal errors of salinity converted from the ^87^Sr/^86^Sr ratios become larger at higher salinity, and the water ^87^Sr/^86^Sr ratios rapidly approached the seawater value above moderate salinity (Figure 4). Shallow mixing curves due to high Sr concentrations of the river water were observed in some estuaries, such as the southern Texas coast in the USA (Walther & Limburg, 2012) and the Avon River in Australia (Bryant et al., 1995), while steep mixing curves due to low Sr concentrations of the river water were observed in other estuaries, such as the San Francisco Estuary in the USA (Hobbs et al., 2010) and the Pampanga River in the Philippines (Bryant et al., 1995). With a shallower mixing curve, we can infer a wider range of salinity. As our mixing curve is relatively steep, we need to be aware of those caveats when using a mixing equation to analyze the habitat salinity of fish.

### 4.2 Otolith ^87^Sr/ ^86^Sr ratios of *Pugitius* sticklebacks

There were significant differences in the otolith ^87^Sr/^86^Sr ratios among the three species of *Pungitius* sticklebacks, suggesting that there were differences in the salinity range of their respective habitats. In addition, comparisons among otolith drilling areas showed that there was a significant difference in the ^87^Sr/^86^Sr ratios between area‐1 (area formed at an early life stage) and area‐3 (area formed at a later life stage) in the freshwater type otoliths. This suggests that the freshwater type in the Shiomi River inhabits low‐salinity environments during the egg or juvenile life stage; however, some individuals move to higher salinity environments as they grow. The ^87^Sr/^86^Sr ratios of area‐1 near the core differed among the three species, suggesting that they use different spawning sites. As most freshwater and brackish‐water types were sampled at the same sites (sites‐8 and 9), our data suggest that past habitat differences in salinity exist between freshwater and brackish‐water types. As *P. tymensis* was only captured at site‐10 during our sampling, and its otolith ^87^Sr/^86^Sr ratios were almost constant among the drilling areas, it is likely that it hardly migrated to sites other than site‐10. Although the otolith ^87^Sr/^86^Sr ratios of the brackish‐water type did not differ between the sampling sites, ^87^Sr/^86^Sr ratios varied greatly among individuals (Figure 5), suggesting that different individuals of the brackish‐water type may utilize habitats with different salinities. We do not know how such great individual variations occur in the brackish‐water type or whether similar patterns can be observed in other rivers.

A mixing equation for water ^87^Sr/^86^Sr ratios and salinity can be used to determine the habitat salinity of fish. For example, Hobbs et al. (2010) used otolith ^87^Sr/^86^Sr ratios and a mixing equation to determine changes in the absolute salinity values of longfin smelt habitats in low‐salinity areas. However, the absolute salinity values calculated from the mixing equation should be interpreted with caution because of the temporal deformation of the mixing equation (see above). Our results of salinity converted from the otolith ^87^Sr/^86^Sr ratios indicate that *P. tymensis* inhabits freshwater (0.08–0.14‰) and the brackish‐water type inhabits a relatively higher salinity range (3.4–6.7‰), with the freshwater type inhabiting the intermediate salinity range (0.53–1.3‰). Significant differences in the otolith ^87^Sr/^86^Sr ratios among the three species could be evident in this study because they inhabited low salinity ranges, as in Hobbs et al. (2010). Because the brackish‐water type had a large error in the converted salinity, caution may be necessary for the interpretation of habitat differences within the brackish‐water type. However, we believe that our conclusions are qualitatively correct. First, we compared individuals collected from the same river simultaneously, indicating that temporal deformation, if any, influences all individuals equally. Therefore, we can conclude that the brackish‐water type uses habitats with relatively higher salinity compared to the other two species and that *P. tymensis* uses habitats with relatively lower salinity than the other two species, and different individuals of the brackish‐water type utilize habitats with different salinities. Second, with regard to the ontogenetic habitat shifts in the freshwater type, the ^87^Sr/^86^Sr ratios of otoliths of the freshwater type, but not those of the brackish‐water type and *P. tymensis*, varied significantly among drilling areas, indicating that they moved within the river (Figure 5).

Arai and Goto (2005, 2008) and Arai et al. (2010) analyzed the Sr/Ca ratios of the otoliths of *Pungitius* sticklebacks from several habitats in Japan and showed that some individuals might migrate among habitats with different salinities. Arai and Goto (2005) analyzed the Sr/Ca ratios of the otoliths of three *Pungitius* species in the Shiomi River and made two observations. The otolith Sr/Ca ratios of the brackish‐water type were significantly higher than those of the other two species, suggesting that the brackish‐water type inhabits areas with a higher salinity range than the other two species. This is consistent with our conclusion based on stable isotope analysis. Additionally, the authors reported that the otolith Sr/Ca ratios of *P. tymensis* and the freshwater type tended to fluctuate with growth. Based on this observation, they suggested that these two species migrated between habitats with varying salinity. However, this interpretation is inconsistent with our observation that *P. tymensis* is sedentary. Arai and Goto (2005) did not base their discussion of otolith Sr/Ca ratios on rearing experimental data; therefore, the fluctuation in their otolith Sr/Ca ratios may be due to other physiological factors, such as elemental uptake influenced by temperature. Our results, based on the otolith ^87^Sr/^86^Sr ratios, may provide more accurate information on habitat use than the otolith Sr/Ca ratios. Furthermore, we demonstrated differences in the habitat salinity range not only within species but also between *P. tymensis* and the freshwater type, which were not detected by the previous otolith Sr/Ca ratio analysis.

Tsuruta et al. (2008) showed that *P. tymensis* and the freshwater type breed in low‐salinity areas compared to the brackish‐water type by directly observing nesting sites in the Shiomi River. Our results further demonstrate that habitat differences in salinity ranges are not limited to the breeding season but also to other life stages and that the overlap in salinity ranges between freshwater and brackish‐water types may increase as they grow older. Ishikawa et al. (2013) showed that the brackish‐water type had the highest salinity tolerance and *P. tymensis* had the lowest salinity tolerance, with the freshwater type being intermediate. Takata (1992) showed that freshwater type fish do not hatch in high‐salinity environments but become more tolerant of high salinity (reduced mortality) as they mature. These results indicated that there is a physiological basis for habitat differentiation among the three species.

### 4.3 Stickleback otolith morphology and development

The two translucent rings and a thin translucent ring were found at almost the same positions on otoliths in all three species (Figure 2; Table A1), suggesting that the growth of otoliths was similar among them. Therefore, these three species may have experienced similar seasonal environmental changes. Several studies have reported sub‐annual rings on the otoliths of the three‐spined stickleback (*Gasterosteus aculeatus*), which belongs to the same family as *Pungitius*, but there is disagreement as to when these sub‐annual rings are formed (Singkam & MacColl, 2018). SingKam and MacColl (2018) concluded that the first opaque rings are formed between birth and the end of the first summer, when food is abundant; the first translucent rings are formed between the end of the first summer and the end of the first winter; and the second opaque rings are formed after the spring of the second year. By applying their interpretation to our results, we can infer that drilling area 1 formed early in the first year, drilling area 2 formed late in the first year, drilling area 3 formed in the second year, and drilling area 4 formed during the third year. Further studies on the formation process of the translucent and opaque rings in stickleback otoliths will not only enhance the interpretation of the results of the elemental analyses of their otoliths but also provide age information useful for studying the evolution of their life history traits.

In this study, the otoliths were polished in the sagittal plane with a thin thickness (Figure 2). We could obtain enough samples for analysis by using this polishing surface. Based on our finding that the thickness of the polished otolith was 133 ± 4 μm, we drilled the otolith to a depth of 40 μm to avoid the inclusion of other areas (Figure 2). However, we cannot exclude the possibility that some areas of the otolith were drilled too deeply because otoliths are not perfectly oval in shape.

## 5 CONCLUSION

Our analysis of the otolith ^87^Sr/^86^Sr ratios clearly showed that the three stickleback species differed in terms of the salinity range of their habitat in a single river. We also demonstrated that individual differences in habitat use could be identified using this method. The use of the otolith ^87^Sr/^86^Sr ratios as a proxy for habitat salinity will be useful not only for sticklebacks but also for other fish species and organisms living in brackish‐water environments.

## AUTHOR CONTRIBUTIONS

**Konomi Uji:** Conceptualization (equal); data curation (lead); formal analysis (lead); funding acquisition (lead); investigation (lead); resources (equal); writing – original draft (lead); writing – review and editing (equal). **Asano Ishikawa:** Data curation (supporting); formal analysis (supporting); resources (equal); writing – review and editing (supporting). **Ki‐Cheol Shin:** Resources (equal); writing – review and editing (supporting). **Ichiro Tayasu:** Funding acquisition (supporting); resources (equal); writing – review and editing (supporting). **Jun Kitano:** Conceptualization (equal); funding acquisition (supporting); resources (equal); writing – original draft (supporting); writing – review and editing (equal).

## CONFLICT OF INTEREST STATEMENT

We have no conflicts of interest associated with this publication.

## Supporting information


Figure S1

Figure S2

Table S1

## Data Availability

All data are included in the Appendix 1 and Supporting Information.

## APPENDIX 1

**TABLE A1:** Fish and otoliths data.

Sample No.	Standard length (mm)	Sampling site	Otolith structure						
			Right or Left	Weight (μg)	Thickness (μm)	Positions from the core toward the edge (μm)			
						Thin translucent ring	Translucent ring‐1	Translucent ring‐2	Edge
BW_1	61.4	6	L	127	–	–	(185)	–	335
BW_2	53.4	6	L	91	–	(111)	(192)	–	307
BW_3	60.0	6	L	108	138	(66)	(186)	(294)	352
BW_4	73.9	8	L	174	–	–	(182)	(307)	384
BW_5	60.5	8	L	99	–	(106)	(198)	–	319
BW_6	52.9	8	L	123	–	91	165	–	295
BW_7	59.8	8	L	105	144	(111)	(171)	–	273
BW_8	60.4	8	L	104	125	(106)	(180)	–	320
BW_9	67.2	8	R	88	107	84	222	–	303
BW_10	56.7	8	R	95	145	–	(183)	–	303
BW_11	52.3	8	R	98	140	–	(184)	–	315
BW_12	51.8	9	L	113	165	(63)	(170)	–	292
BW_13	59.5	9	L	118	139	–	–	–	352
BW_14	56.5	9	L	137	132	100	181	–	303
BW_15	60.7	9	L	137	108	–	142	297	396
Mean of BW	59.1			115	134	92	178	297	324
FW_1	54.5	8	L	120	–	–	(157)	–	305
FW_2	56.4	8	L	131	–	–	195	–	334
FW_3	52.7	9	L	112	–	110	158	–	288
FW_4	63.8	9	L	160	–	–	177	298	373
FW_5	40.4	9	L	62	–	–	(184)	–	260
FW_6	52.7	9	L	99	144	–	(122)	–	279
FW_7	46.4	9	L	69	110	–	127	–	299
Mean of FW	52.4			108	127	110	164	298	324
Ptym_1	68.5	10	L	208	–	(92)	(181)	(289)	392
Ptym_2	68.1	10	L	241	–	–	–	(294)	415
Ptym_3	74.6	10	L	183	–	–	(205)	(331)	398
Ptym_4	66.2	10	L	251	–	–	(169)	(303)	448
Ptym_5	59.2	10	L	193	120	–	162	281	394
Ptym_6	67.0	10	L	177	123	–	(181)	(303)	396
Ptym_7	58.2	10	L	200	134	126	207	341	420
Ptym_8	56.3	10	R	183	123	–	150	296	386
Ptym_9	59.4	10	R	188	160	–	(151)	(298)	386
Mean of Ptym	64.2			203	132	126	173	306	400
Mean of all	59.1			135	133	102	171	303	345

*Note*: Right (R) or left (L) otoliths were extracted from each fish. The positions of the translucent rings and thicknesses of the polished otoliths were measured. Otoliths whose rings or thickness were difficult to be measured were marked as “–”. The positions of the clearly observable translucent rings are shown without parentheses. When the translucent rings were wide, we measured their positions on the outermost part. The positions of the rings that were observed only vaguely are shown in parentheses. Mean values were calculated using values without parentheses.

Abbreviations: BW, brackish‐water type; FW, freshwater type; Ptym, *Pungitius tymensis*.

**TABLE A2:** ^87^Sr/^86^Sr ratios of otoliths from each drilling area.

Sample No.	^87^Sr/^86^Sr ratios of otoliths							
	Area‐1	SE	Area‐2	SE	Area‐3	SE	Area‐4	SE
BW_1	–	–	–	–	–	–	–	–
BW_2	0.70777	2.3 × 10^−5^	0.70769	2.4 × 10^−5^	0.70816	1.9 × 10^−5^	–	–
BW_3	0.70900	2.8 × 10^−5^	0.70873	2.3 × 10^−5^	0.70880	2.4 × 10^−5^	–	–
BW_4	–	–	–	–	–	–	–	–
BW_5	–	–	–	–	–	–	–	–
BW_6	–	–	–	–	–	–	–	–
BW_7	0.70913	2.2 × 10^−5^	0.70918	2.3 × 10^−5^	0.70922	2.3 × 10^−5^	–	–
BW_8	0.70805	2.1 × 10^−5^	0.70805	1.7 × 10^−5^	0.70880	2.2 × 10^−5^	–	–
BW_9	0.70890	2.1 × 10^−5^	0.70866	2.3 × 10^−5^	0.70901	2.5 × 10^−5^	–	–
BW_10	0.70806	3.0 × 10^−5^	0.70820	1.9 × 10^−5^	0.70888	2.7 × 10^−5^	–	–
BW_11	0.70823	2.5 × 10^−5^	0.70838	2.1 × 10^−5^	0.70866	2.2 × 10^−5^	–	–
BW_12	–	–	–	–	–	–	–	–
BW_13	0.70904	2.2 × 10^−5^	0.70890	2.0 × 10^−5^	0.70903	2.3 × 10^−5^	–	–
BW_14	0.70757	2.4 × 10^−5^	0.70759	1.9 × 10^−5^	0.70832	2.2 × 10^−5^	–	–
BW_15	–	–	–	–	–	–	–	–
Mean of BW	0.70842		0.70838		0.70876			
FW_1	0.70677	3.6 × 10^−5^	0.70762	1.9 × 10^−5^	0.70864	2.5 × 10^−5^	–	–
FW_2	–	–	–	–	–	–	–	–
FW_3	–	–	–	–	–	–	–	–
FW_4	0.70692	2.9 × 10^−5^	0.70694	2.2 × 10^−5^	0.70804	2.5 × 10^−5^	–	–
FW_5	0.70694	2.1 × 10^−5^	0.70705	2.2 × 10^−5^	0.70714	2.4 × 10^−5^	–	–
FW_6	0.70667	2.0 × 10^−5^	0.70680	2.2 × 10^−5^	0.70712	2.2 × 10^−5^	–	–
FW_7	0.70663	2.1 × 10^−5^	0.70670	2.3 × 10^−5^	0.70699	2.2 × 10^−5^	–	–
Mean of FW	0.70678		0.70702		0.70759			
Ptym_1	–	–	–	–	–	–	–	–
Ptym_2	0.70573	3.0 × 10^−5^	0.70588	2.5 × 10^−5^	0.70586	2.1 × 10^−5^	0.70606	1.9 × 10^−5^
Ptym_3	0.70598	2.0 × 10^−5^	0.70617	2.4 × 10^−5^	0.70611	2.2 × 10^−5^	–	–
Ptym_4	–	–	–	–	–	–	–	–
Ptym_5	0.70595	2.2 × 10^−5^	0.70595	2.0 × 10^−5^	0.70591	2.0 × 10^−5^	0.70589	2.2 × 10^−5^
Ptym_6	0.70580	2.4 × 10^−5^	0.70591	2.3 × 10^−5^	0.70606	2.3 × 10^−5^	0.70592	1.9 × 10^−5^
Ptym_7	0.70579	2.6 × 10^−5^	0.70588	2.7 × 10^−5^	0.70607	2.1 × 10^−5^	0.70607	2.0 × 10^−5^
Ptym_8	–	–	–	–	–	–	–	–
Ptym_9	–	–	–	–	–	–	–	–
Mean of Ptym	0.70585		0.70596		0.70600		0.70598	
Mean of all	0.70731		0.70738		0.70773		0.70598	

Abbreviations: BW, brackish‐water type; FW, freshwater type; Ptym, *Pungitius tymensis*.

**TABLE A3:** Water data. The salinity of the water was measured by converting its conductivity.

Sampling site	Sampling date	Salinity (‰)	^87^Sr/^86^Sr ratios of water	SE
Site‐1	May 2016	31.1	0.70916	5.3 × 10^−6^
	Morning of July 2016	30.5	0.70918	5.7 × 10^−6^
	Afternoon of July 2016	28.0	0.70917	5.6 × 10^−6^
	May 2018	31.1	0.70915	7.9 × 10^−6^
Site‐2	May 2016	19.6	0.70911	5.5 × 10^−6^
	Morning of July 2016	6.0	0.70891	5.4 × 10^−6^
	Afternoon of July 2016	28.2	0.70916	4.7 × 10^−6^
	May 2018	29.3	0.70914	8.8 × 10^−6^
Site‐3	May 2016	13.0	0.70901	6.0 × 10^−6^
	Morning of July 2016	5.8	0.70892	5.5 × 10^−6^
	Afternoon of July 2016	27.9	0.70918	5.5 × 10^−6^
	May 2018	27.9	0.70915	7.3 × 10^−6^
Site‐4	May 2016	9.6	0.70891	5.0 × 10^−6^
	Morning of July 2016	5.5	0.70890	6.1 × 10^−6^
	Afternoon of July 2016	6.0	0.70890	5.3 × 10^−6^
	May 2018	27.1	0.70913	7.6 × 10^−6^
Site‐5	May 2016	9.1	0.70891	5.6 × 10^−6^
	Morning of July 2016	5.3	0.70888	5.5 × 10^−6^
	Afternoon of July 2016	6.2	0.70891	4.6 × 10^−6^
	May 2018	24.6	0.70911	8.4 × 10^−6^
Site‐6	May 2016	1.4	0.70767	5.8 × 10^−6^
	Morning of July 2016	1.3	0.70799	5.3 × 10^−6^
	Afternoon of July 2016	0.4	0.70692	5.3 × 10^−6^
	May 2018	16.0	0.70900	9.5 × 10^−6^
Site‐7	May 2016	1.4	0.70761	5.3 × 10^−6^
	Morning of July 2016	1.0	0.70772	5.5 × 10^−6^
	Afternoon of July 2016	0.3	0.70670	5.2 × 10^−6^
	May 2018	17.6	0.70902	8.7 × 10^−6^
Site‐8	May 2016	1.1	0.70786	6.0 × 10^−6^
	Morning of July 2016	0.3	0.70659	5.9 × 10^−6^
	Afternoon of July 2016	0.3	0.70648	5.5 × 10^−6^
	May 2018	1.2	0.70681	6.4 × 10^−6^
Site‐9	May 2016	0.9	0.70674	5.7 × 10^−6^
	Morning of July 2016	0.3	0.70652	5.4 × 10^−6^
	Afternoon of July 2016	0.1	0.70600	6.0 × 10^−6^
	May 2018	1.0	0.70677	9.0 × 10^−6^
Site‐10	May 2016	0.2	0.70599	5.0 × 10^−6^
	Morning of July 2016	0.1	0.70574	5.5 × 10^−6^
	Afternoon of July 2016	0.1	0.70572	5.6 × 10^−6^
	May 2018	0.8	0.70643	8.4 × 10^−6^
