# Supplementary material for: Strontium isotope analysis of otoliths reveals differences in the habitat salinity among three sympatric stickleback species of the genus Pungitius
Source: Ecol Evol. 2023 Sep 3;13(9):e10463. doi: 10.1002/ece3.10463 (PMC10475353; doi:10.1002/ece3.10463)
Supplement: Supplementary file 1 — Figure S1 [file ECE3-13-e10463-s003.docx]

**Supplementary Figure 1:** Result of STRUCTURE analysis. Different colors indicate different genetic ancestries. A single column indicates a single individual. Based on a previous study (Ishikawa et al. 2013), the red and green colors indicate the freshwater type and the brackish-water type, respectively. A subset of the brackish-water type was used for the otolith analysis.

**
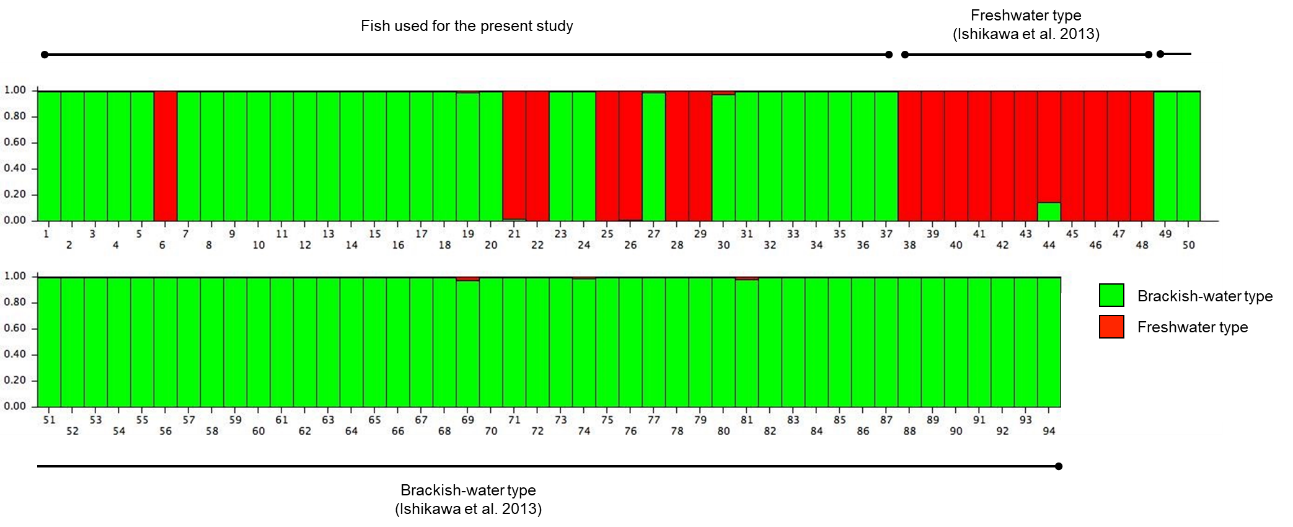
**
