# Supplementary material for: Strontium isotope analysis of otoliths reveals differences in the habitat salinity among three sympatric stickleback species of the genus Pungitius
Source: Ecol Evol. 2023 Sep 3;13(9):e10463. doi: 10.1002/ece3.10463 (PMC10475353; doi:10.1002/ece3.10463)
Supplement: Supplementary file 2 — Figure S2 [file ECE3-13-e10463-s002.docx]

**Supplementary Figure 2**: Geological information on the Shiomi River system. The left panel indicates a map created by processing GSI Tile (elevation tile) (Geospatial Information Authority of Japan, 2017). The right panel indicates a map created by processing the Seamless Digital Geological Map of Japan (1:200,000) (Geological Survey of Japan, AIST, 2022).


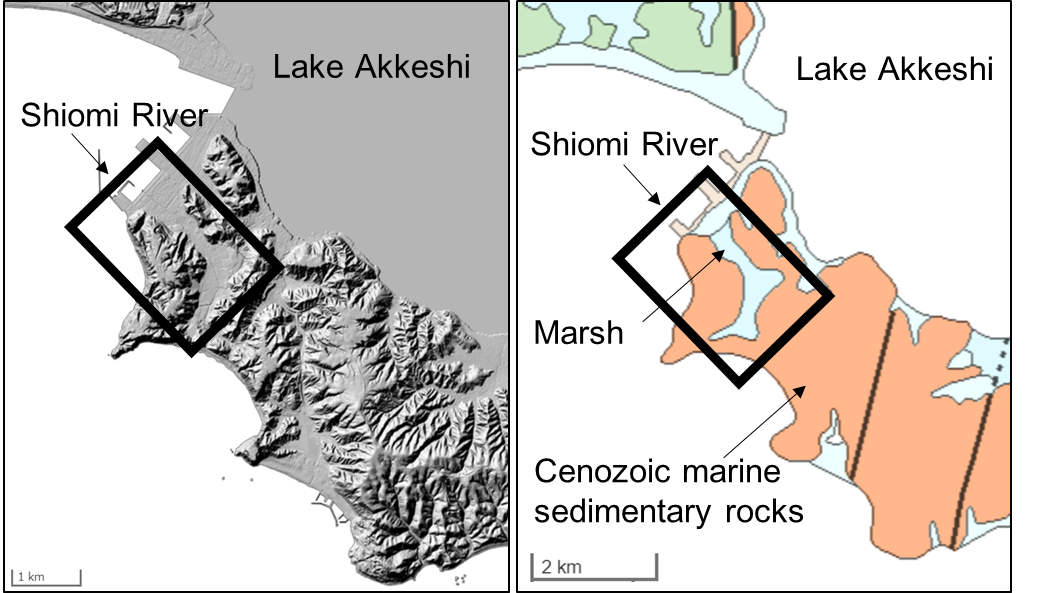


References

Geospatial Information Authority of Japan, (2017), GSI Tile (elevation tile). https://maps.gsi.go.jp/development/ichiran.html [Accessed: December 14, 2022]

Geological Survey of Japan, AIST (2022), Seamless digital geological map of Japan V2 1:200,000. <https://gbank.gsj.jp/seamless> [Accessed: December 14, 2022]
